# Editorial to the Organophosphorus Chemistry Special Issue of Molecules (2012–2014)

**DOI:** 10.3390/molecules191015408

**Published:** 2014-09-26

**Authors:** György Keglevich

**Affiliations:** Department of Organic Chemistry and Technology, Budapest University of Technology and Economics, 1521 Budapest, Hungary; E-Mail: gkeglevich@mail.bme.hu; Tel.: +36-1-463-1111 (ext. 5883); Fax: +36-1-463-3648

The review entitled “Organophosphorus Chemistry for the Synthesis of Dendrimers” gives an overview of the methods of synthesis of phosphorus-containing dendrimers, with emphasis on the various roles played by the chemistry of phosphorus [1]. It is demonstrated that the presence of phosphorus atom(s) at each branching point of the dendrimeric structure is particularly important and highly valuable.

The review “Stoichiometric and Catalytic Synthesis of Alkynylphosphines summarizes the possibilities for the preparation of alkynylphosphines that, or their borane complexes, are available either through C–P bond forming reactions, or through modification of the phosphorus or the alkynyl function of various alkynyl phosphorus derivatives [2]. The latter strategy involving phosphoryl reduction is the method of choice for preparing primary and secondary alkynylphosphines, while the former strategy is employed for the synthesis of tertiary alkynylphosphines, or their borane complexes. Recently efficient catalytic procedures, involving copper complexes and either an electrophilic or a nucleophilic phosphorus reagent have emerged.

New developments of the Kabachnik–Fields (K-F) reaction were surveyed in the review “The Kabachnik-Fields Reaction: Mechanism and Synthetic Use” [3]. The monitoring of a few K–F reactions by *in situ* Fourier transform IR spectroscopy has indicated the involvement of the imine intermediate that was also justified by theoretical calculations. The K–F reaction was extended to >P(O)H species, comprising cyclic phosphites, acyclic and cyclic *H*-phosphinates, as well as secondary phosphine oxides. The synthesis under solvent-free microwave conditions is a good method of choice, as sophisticated and environmentally unfriendly catalysts suggested by literature methods are completely unnecessary under microwave conditions. The double K–F reaction made available bis(phosphonomethyl)amines.

The paper “Hydrophosphonylation of Nanoparticle Schiff Bases as a Mean for Preparation of Aminophosphonate-Functionalized Nanoparticles” describes results on magnetic nanoparticles with a modified surface that are attractive alternatives to deliver therapeutic agents [4]. The surface of the iron oxide nanoparticles was modified with aminophosphonic acids by applying the classical hydrophosphonylation protocol.

Recent achievements in the field of phosphinic dipeptide derivatives bearing appropriate side-chain substituents are summarized in the review entitled “Synthesis and Modifications of Phosphinic Dipeptide Analogues” [5]. Improved methods for the formation of the phosphinic peptide backbone, including stereoselective and multicomponent reactions, are presented. Parallel modifications leading to the structurally diversified substituents are also described, and selected examples of the biomedical applications of the title compounds are given.

In the paper “Chemistry of Phosphorylated Formaldehyde Derivatives. Part I.” a few structurally related compounds, such as thioacetals, aminonitriles, aminomethylphosphinoyl compounds, are discussed [6]. The halogen aminals and acetals of phosphorylated formaldehyde, and a phosphorylated gem-diol of formaldehyde were discussed separately.

The chemical synthesis of DNA and RNA is usually carried out using nucleoside phosphoramidites or *H*-phosphonates as synthons. The review “Synthesis of DNA/RNA and their Analogs via Phosphoramidite and *H*-Phosphonate Chemistries” focuses on the phosphorus chemistry behind these synthons, and how it has been developed [7]. Additionally, the synthesis and properties of certain DNA and RNA analogues that are modified at the phosphorus atom were also discussed. These analogues include boranephosphonates, metallophosphonates, and alkylboranephosphines.

The research “Cesium Ion Removal from Aqueous Solutions through Adsorption onto Florisil^®^ Impregnated with Trihexyl(tetradecyl)phosphonium Chloride” describes the adsorption performance of Florisil^®^ impregnated with trihexyl(tetradecyl)phosphonium chloride in the process of cesium ion removal from aqueous solutions [8]. The adsorption process has been investigated as a function of pH, solid:liquid ratio, adsorbate concentration, contact time, and temperature.
